# Are genes faster than crabs? Mitochondrial introgression exceeds larval dispersal during population expansion of the invasive crab *Carcinus maenas*

**DOI:** 10.1098/rsos.140202

**Published:** 2014-10-15

**Authors:** John A. Darling, Yi-Hsin Erica Tsai, April M. H. Blakeslee, Joe Roman

**Affiliations:** 1 National Exposure Research Laboratory, United States Environmental Protection Agency, 109 TW Alexander Drive, Durham, NC 27711, USA; 2 Department of Ecology and Evolutionary Biology, University of Colorado, Boulder, CO 80309, USA; 3 Department of Biology, Long Island University, 720 Northern Boulevard, Brookville, NY 11548, USA; 4 Gund Institute for Ecological Economics, University of Vermont, Burlington, VT 05405, USA

**Keywords:** introgression, admixture, invasive species, range expansion

## Abstract

Biological invasions offer unique opportunities to investigate evolutionary dynamics at the peripheries of expanding populations. Here, we examine genetic patterns associated with admixture between two distinct invasive lineages of the European green crab, *Carcinus maenas* L., independently introduced to the northwest Atlantic. Previous investigations based on mitochondrial DNA sequences demonstrated that larval dispersal driven by advective currents could explain observed southward displacement of an admixture zone between the two invasions. Comparison of published mitochondrial results with new nuclear data from nine microsatellite loci, however, reveals striking discordance in their introgression patterns. Specifically, introgression of mitochondrial genomes relative to nuclear background suggests that demographic processes such as sex-biased reproductive dynamics and population size imbalances—and not solely larval dispersal—play an important role in driving the evolution of the genetic cline. In particular, the unpredicted introgression of mitochondrial alleles against the direction of mean larval dispersal in the region is consistent with recent models invoking similar demographic processes to explain movements of genes into invading populations. These observations have important implications for understanding historical shifts in *C. maenas* range limits, and more generally for inferences of larval dispersal based on genetic data.

## 2. Introduction

Population dynamics at species range limits can provide evolutionary insights into phenomena as varied as adaptation, speciation, range expansion, and population distribution and persistence [1–6]. Because biological invasions can be tracked in real time, they may provide unprecedented opportunities for revealing these dynamic processes as they occur [7]. Interactions between invading and established populations (or between multiple invasive populations) can be particularly revealing, delivering novel insights into the dynamics of genetic introgression and admixture, gene flow and dispersal [8–10]. Recent simulation studies suggest that demographic processes during range expansions can drive selectively neutral, stochastic shifts in allele frequency, providing a general mechanism for extensive genetic introgression across population or species boundaries [11,12]. These processes may have somewhat unexpected outcomes. For instance, a neutral model developed by Currat *et al.* [13] predicts dramatic introgression of genes from demographically stable, established populations into rapidly expanding, invasive populations. Examinations of the spatio-temporal patterns of genetic variation associated with biological invasions may enable direct tests of such theoretical predictions, thus shedding light on processes underlying colonization dynamics and genetic exchange among populations.

**Figure 1.**
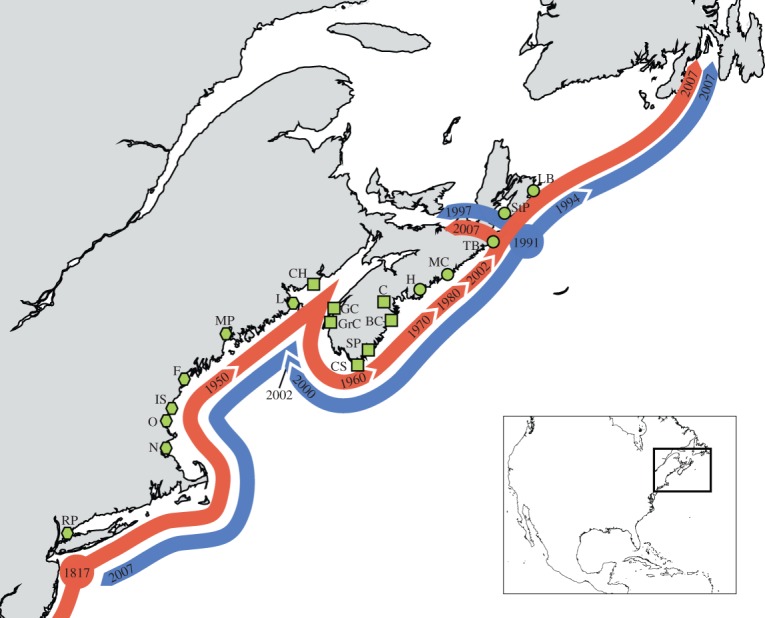
Distribution of sampling sites and range expansion of mitochondrial haplotypes of *C. maenas*. For the purposes of genetic diversity analyses, sampling sites (green) have been divided into southern (hexagons), northern (circles) and central (squares) regions; the latter corresponds to the primary admixture zone. Full locality names and additional details can be found in table 1. Arrows show the spread of the two mitochondrial haplotypes (red = southern; blue = northern) through time. Large circles give the likely sites where *C. maenas* was first introduced. Adapted from [19–21]. Arrows are meant to indicate geographical extent of haplotype clusters only, and not actual movement of haplotypes; for instance, simultaneous arrival of southern and northern haplotype clusters to Newfoundland in 2007 has been ascribed to anthropogenic translocation from the admixture zone near Halifax [20].

While empirical investigations of such dynamics in natural systems are becoming more common [8,10,14], they remain rare for marine taxa [15]. Here, we describe genetic analysis of a marine system well suited to test the applicability of these neutral models for stochastic shifts in allele frequency driven by demographic processes. The European green crab, *Carcinus maenas*, one of the most notorious global marine invaders, was first introduced to the northwest Atlantic in the early nineteenth century [16,17], where it spread northwards along the coastline, eventually reaching a stable northern range limit near Halifax, Nova Scotia, by the 1970s [18,19]. In the late 1980s, a novel introduction resulted in a second cryptic invasion from Europe to northern Nova Scotia that expanded rapidly, achieving high densities along the Nova Scotian coast by the 2000s (figure 1) [19]. This new invasion derives from native northern European sources genetically divergent from the earlier invasion, and genetic studies of the system confirmed the presence of an admixture zone initially centred near the point of contact in central Nova Scotia [19–21]. Using mitochondrial haplotype frequency distributions, Pringle *et al.* [21] explored the temporal dynamics of this system with a larval transport model based on coastal advective currents. Their model was able to predict the evolution of a mitochondrial frequency cline between southern and northern populations, revealing southward displacement of the cline consistent with predominant current patterns in the region.

The dynamic admixture of two independently introduced *C. maenas* populations offers a remarkably tractable system for investigating evolutionary dynamics at population boundaries, the mechanisms of genetic introgression and the demographic processes underlying these phenomena. We expand here on earlier work by introducing analysis of nuclear microsatellite data, revealing unexpected discordance in the temporal dynamics of mitochondrial and nuclear genomes across the *C. maenas* admixture zone. These patterns suggest an important role for demographic processes other than larval dispersal in shaping the evolution of genetic clines and offer novel support for hypotheses explaining *C. maenas* range limits in the north Atlantic.

## 3. Material and methods

### 3.1 Molecular methods

Crab samples, tissues and mitochondrial cytochrome *c* oxidase subunit I (COI) gene sequences used in this study have been described elsewhere [20]. Figure 1 and table 1 provide details on sampling locations. A 502 bp fragment of the COI gene amplified with primers and protocols designed for *Carcinus* [22] was used for all sequence analyses; COI sequence data are a subset of those used in Pringle *et al.* [21]. Microsatellite loci Cma01EPA, Cma02EPA, Cma03EPA, Cma04EPA, Cma05EPA, Cma08EPA, Cma09EPA, Cma14EPA and Cma16EPA were amplified from genomic DNA using previously described molecular protocols [17,23]. Samples that failed to amplify at three or more loci after two attempts were dropped from the dataset.

**Table 1.** Sampling site details. Distance is along-shore distance from the southernmost site at Rye Playland, NY. *N* is the number of individuals for which data was collected at either mitochondrial or microsatellite loci.

					*N* (mitochondrial)			*N* (microsatellite)		
sample location	sample ID	distance (km)	latitude (°)	longitude (°)	2000	2002	2007	2000	2002	2007
Rye Playland, NY	RP	0	41.00	−73.57	—	9	12	—	9	12
Nahant, MA	N	572	42.60	−70.65	—	21	19	—	21	19
Odiorne, NH	O	612	43.00	−70.73	—	—	17	—	—	17
Isle of Shoals, NH	IS	619	42.98	−70.60	—	19	20	—	19	20
Falmouth, ME	F	708	43.70	−70.22	—	18	16	—	18	16
Moosepoint, ME	MP	982	44.45	−69.92	15	—	—	15	—	—
Lubec, ME	L	1258	44.08	−66.98	5	—	7	5	—	8
Chance Harbor, NB	CH	1346	45.12	−66.98	4	20	—	4	20	—
Gulliver's Cove, NS	GC	1920	44.48	−66.08	8	20	19	8	20	20
Grosses Coques, NS	GrC	1936	44.35	−66.10	8	—	—	9	—	—
Cape Sable, NS	CS	2088	44.43	−66.58	12	19	15	12	19	15
Sandy Point, NS	SP	2135	43.68	−65.24	11	15	—	12	15	—
Broad Cove, NS	BC	2216	44.15	−64.48	7	21	—	7	21	—
Chester, NS	C	2286	44.53	−64.57	9	19	—	9	19	—
Halifax, NS	H	2370	44.62	−63.55	12	20	17	12	20	21
Murphy's Cove, NS	MC	2480	44.95	−62.07	9	20	17	9	21	20
Tor Bay, NS	TB	2531	45.18	−61.35	10	—	—	10	—	—
St Peter's, NS	StP	2644	45.65	−60.87	—	20	22	—	20	23
Louisborg, NS	LB	2705	45.92	−59.95	10	—	—	10	—	—

### 3.2 Genetic analysis

Standard genetic diversity indices were generated for all samples using Arlequin v. 3.0 [24] for mitochondrial data and Fstat v. 2.9.3.2 [25] for microsatellite data. Significant differences (*α*=0.05) in mean diversity between southern, central and northern sample regions were determined using the Tukey–Kramer method to account for unequal sample sizes, implemented in the R statistical programming environment v. 3.0.2 (http://www.R-project.org). Regional boundaries were drawn in the south between Lubec, ME (L) and Chance Harbor, NB (CH) and in the north between Chester, NS (C) and Halifax, NS (H) (figure 1). These regions correspond roughly to the southern and northern limits of the observed nuclear admixture zone in the earliest temporal samples. Analyses were run separately on each year's data (i.e. 2000, 2002 and 2007). Comparisons between mitochondrial and nuclear loci were not made with the data from the 2000 collection due to limited sampling in the southern region in that year.

To assess nuclear admixture, we adopted the Bayesian inference model implemented in Structure v. 2.3.4 [26] to assign individual crab microsatellite genotypes to population clusters. Models were run independently for each collection year. Initial runs with the number of population clusters ranging from *K*=1 to *K*=*n* (where *n* was the total number of sample sites for that year) found that the optimal number of population clusters was *K*=2 in all collection years, corresponding to the northern and southern invasion (not shown). To assess individual admixture, subsequent model runs were conducted with *K*=2 (five runs of 10^4^ generations burn-in followed by 10^5^ generations of data collection) and admixture between the two clusters was estimated using the individual co-ancestry coefficient *Q* of the highest likelihood model run. All analyses assumed uncorrelated allele frequencies allowing for admixture.

Effect of geography on admixture was determined based on changes in allele frequency against along-shore linear distance from the southernmost sampling site at Rye Playland, NY (RP). Distances were calculated with ArcGIS (ESRI, Redlands, CA, USA). Genetic clines consistent with neutral assumptions were assessed using a Metropolis–Hastings Markov chain Monte Carlo (MCMC) algorithm implemented in the HZAR R package [27]. The HZAR algorithm takes both allele frequencies and sample sizes into account, and maximum-likelihood clines and 95% intervals for cline centre and cline width were determined separately at both nuclear and mitochondrial loci for years 2002 and 2007. Limited sampling in the southern portion of the range in 2000 prevented direct comparison of cline shapes across all years. The MCMC was run for 10^6^ iterations with 2.5×10^5^ discarded as burn-in. Clines were based on frequency of southern haplotypes in the case of mitochondrial data and on mean population co-ancestry (*Q*) in the southern Structure cluster in the case of microsatellite date.

To identify individuals with cytonuclear discordance, we conducted additional Structure runs employing the USEPOPINFO flag to pre-assign individuals to either the northern or the southern mitochondrial haplotype cluster. Crabs identified by Structure as ‘immigrants’ in this analysis suggest individuals in which northern mitochondrial haplotypes have been introduced into southern nuclear backgrounds or vice versa.

## 4. Results

### 4.1 Genetic diversity

**Figure 2.**
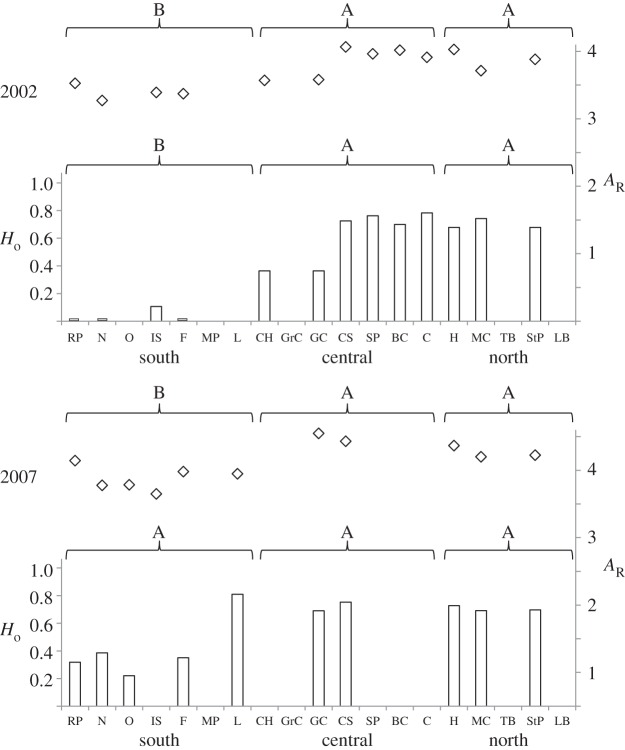
Comparisons of genetic diversity measures across three regions in 2002 and 2007 of *C. maenas*. Nuclear microsatellite allelic richness (*A*_*R*_, right axis, open diamonds) and mitochondrial gene diversity (*H*_*o*_, left axis, columns) are plotted for all sampling sites. Sites without bars were unsampled in that year. Regions with different letters have significantly different mean diversity (*α*=0.05).

In 2002, genetic diversity was significantly lower in the southern part of the range (points from Lubec, ME (L) south) for both nuclear and mitochondrial loci (figure 2). By 2007, this regional difference in mitochondrial gene diversity had eroded (i.e. non-significant pairwise differences across all regions). However, there remained a significant deficiency in nuclear allelic richness in the southern region.

### 4.2 Introgression patterns

**Figure 3.**
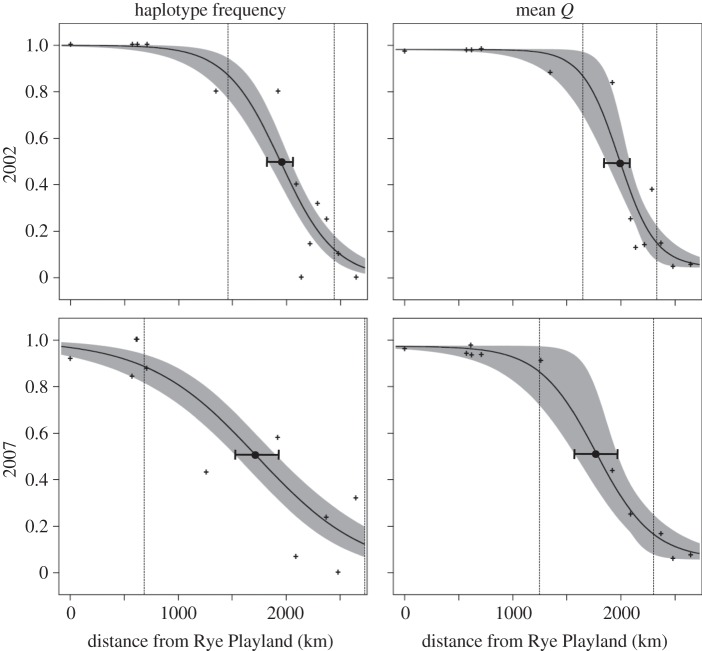
Plots of maximum-likelihood clines and their associated fuzzy cline regions (95% credible intervals) for mitochondrial DNA and microsatellite data of *C. maenas*. Also indicated are maximum-likelihood estimates of cline centre (dots) with two log-likelihood low and high estimates (whiskers). Dashed vertical lines show maximum-likelihood estimates for cline width.

Genetic cline analysis reveals shifts in both nuclear and mitochondrial clines over the period between 2002 and 2007 (figure 3 and table 2). Cline centres showed similar southward shifts for both marker systems. In 2002, maximum-likelihood estimates for cline centre were 1981 km for nuclear loci (measured from Rye Playland, NY, the southernmost site) and 1943 km for mitochondrial COI. By 2007, those cline centres had shifted south by 213 km for nuclear loci and by 214 km for the mitochondrial locus. By contrast, cline width changed dramatically for the mitochondrial locus, with maximum-likelihood estimates more than doubling from 1009 km (95% CI, 749–1376 km) to 2027 km (95% CI, 1584–2692 km), while exhibiting only a very small shift for microsatellite loci (95% confidence intervals were largely overlapping from 329–1115 km in 2002 to 352–1613 km in 2007; table 2).

**Table 2.** Maximum-likelihood (ML) estimates of 95% intervals for cline centre and cline width based on nuclear microsatellite and mitochondrial COI data.

		cline centre			cline width		
	year	95% low	ML estimate	95% high	95% low	ML estimate	95% high
microsatellite	2002	1837	1981	2076	329	672	1115
	2007	1535	1768	1978	352	1058	1613
COI	2002	1819	1943	2043	749	1009	1376
	2007	1522	1729	1937	1584	2027	2692

**Figure 4.**
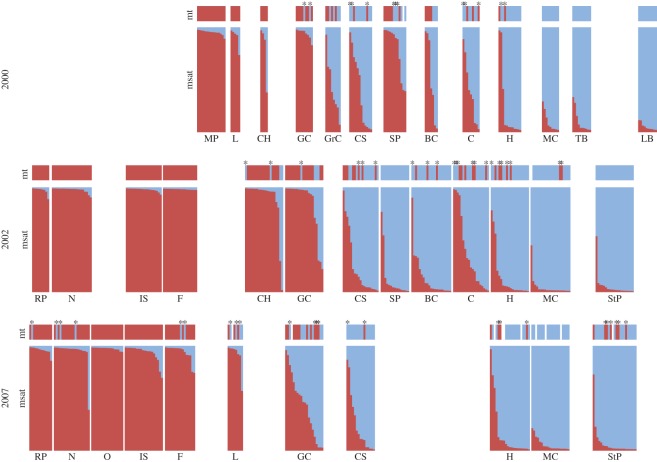
Introgression patterns in *C. maenas*. Each set of two blocks is a sampling population; sample site IDs are indicated under the blocks. Sampling populations are arranged from the southernmost site on the left (Rye Playland, RP) to the northernmost site on the right (Louisbourg, LB) and are arranged so that they line up across sampling years (top: 2000; middle: 2002; bottom; 2007). Each block is composed of vertical bars, with each bar representing an individual crab. All bars are the same width, so blocks are scaled to sample size. The upper block represents the mitochondrial DNA haplotype: red for southern haplotype, blue for northern and white for missing data. The lower block shows the co-ancestry coefficient (*Q*) based on microsatellite data as determined by Structure analysis. Red represents the proportion of the nuclear genome within the southern microsatellite cluster; blue is the proportion in the northern cluster. Individuals are sorted by *Q* within sampling groups. Stars indicate individuals with cytonuclear mismatches—i.e. northern COI haplotype in southern nuclear background or vice versa. Cytonuclear mismatches were identified using Structure analysis as explained in the main text.

Analysis at the level of individual genotypes reveals the geographical extent of admixed crabs for the three collection periods (figure 4). In 2000, admixture was limited primarily to the region between southern New Brunswick (CH) and Halifax, NS (H), as indicated by the presence of crabs exhibiting both nuclear admixture (mixed co-ancestry between southern and northern microsatellite clusters) and cytonuclear mismatches (southern COI haplotypes observed in northern nuclear backgrounds, or vice versa). By 2007, the nuclear admixture zone had broadened somewhat, primarily expanding to the south as revealed by admixed genotypes at Lubec, ME (L) and modest increases in proportional co-ancestry in the northern microsatellite cluster as far south as Nahant, MA (N). Analysis of cytonuclear discordance in 2007 reveals northern COI haplotypes in crabs belonging to the southern nuclear cluster as far south as Rye Playland, NY (RP, the southernmost collection site), whereas southern COI haplotypes were observed in crabs with northern nuclear background at the northernmost collection site at St Peter's, NS (StP). At nuclear loci, extensive introgression of southern alleles appears to be limited mainly to Halifax and points south, even in 2007 (although one individual at St Peter's did exhibit a nuclear genotype consistent with co-ancestry predominantly in the southern cluster).

## 5. Discussion

The unexpected discordance between patterns of genetic variation at nuclear and mitochondrial loci strongly suggests that larval transport is not the only factor shaping temporal dynamics of genetic variation in this system. Specifically, our analyses indicate that the introgression of mitochondrial genomes from the southern to the northern populations, against the direction of mean larval transport and beyond a previously established range limit for *C. maenas*, may be explained in part by underlying demographic processes.

An earlier examination of mitochondrial haplotype frequencies of *C. maenas* in the northwest Atlantic [21] observed a pronounced southward displacement in the mitochondrial haplotype frequency cline observed in this study (figure 3). This temporal shift was shown to be consistent with larval dispersal driven by predominantly southward advective coastal currents in the region, and larval transport models accurately predicted the evolution of the mitochondrial genetic cline over time. In this study, the southward expansion of northern haplotypes is reflected in a 213 km southward shift in the mitochondrial cline centre between 2002 and 2007, as well as significant elevation of mitochondrial gene diversity in the southern portion of the range in the same time period (figure 2). Similarly, the shift in the nuclear genetic cline is consistent with an overall displacement of the admixture zone to the south (figure 3). However, microsatellite data revealed only a modest broadening of cline width compared with the mitochondrial data (figure 3), and there was no significant increase in microsatellite allelic richness in the southern part of the range beyond the primary admixture zone in western Nova Scotia and New Brunswick (figure 2). Assuming that sex-biased larval dispersal is highly unlikely for *C. maenas*, advective currents would be expected to drive similar shifts in both nuclear and mitochondrial cline centres, consistent with our observations. However, the relatively rapid bi-directional expansion of the mitochondrial cline suggests an important role for demographic processes that, unlike larval dispersal, are expected to have differential influences on nuclear and mitochondrial genomes.

Discordance between genomes is revealed most strikingly by patterns of cytonuclear mismatch in individual crabs. Nuclear admixture between northern and southern genetic clusters remains largely constrained to the primary admixture zone, with some modest admixture in samples from northern New England (figure 4). In stark contrast, mitochondrial introgression appears to have occurred across the entire range by 2007, resulting in large numbers of crabs with cytonuclear mismatches in populations at both the southern and northern peripheries. These changes are highly unlikely to be sampling artefacts. For instance, if we assume that the true haplotype frequency actually remained unchanged between 2002 and 2007 at the northernmost site (StP; proportion of southern haplotypes *p*=7/22 in 2007) then the probability of making the observation of zero southern haplotypes at that site in 2002 is *pbinom* (*x*=0, *n*=20, *p*=7/22)=0.00047.

The bidirectional expansion of cytonuclear mismatches is particularly conspicuous given the overall shift in cline centre to the south. Notably, from 2001 to 2007, Pringle *et al*. [21] documented spread of southern mitochondrial haplotypes (i.e. those with previous northern range limits near Halifax, NS [22,23]) towards the northeast and against the direction of advective currents. They accounted for this unanticipated result by introducing into their model substantial variation in larval transport (i.e. *L*_*diff*_), which would allow larval dispersal northward and expansion of the southern mitochondrial haplotypes. However, the magnitude of *L*_*diff*_ was roughly 3.5 times greater than mean larval transport and substantially higher than what would be predicted by known regional current patterns. The authors suggested that variation in larval dispersal may have been shaped by long-distance anthropogenic transport or by differences in currents encountered by larval cohorts spawned at different times.

A number of recent theoretical and empirical studies reveal that demographic dynamics at expanding range margins may drive surprisingly large stochastic shifts in allele frequency [2]. Rapid population expansions can lead to genetic ‘surfing’ of alleles at the expansion front, often leading to dramatic genetic differentiation between peripheral and core populations [11,12]. In cases of contact between established and newly expanding populations, these dynamics can result in extensive genetic introgression across population or even species boundaries [15,28,29]. Particularly interesting in this regard are recent simulations demonstrating dramatic introgression driven by demographic imbalances between rapidly expanding invasive populations and relatively static, established ones [13]. Under these circumstances, initial crosses in the contact zone are likely to be interpopulational, as the first few invading individuals are unlikely to find mates from their own population given its low density. Thereafter, genes from the established population that became introgressed into the invading population via interbreeding would experience subsequent amplification through rapid demographic expansion at the invasion front, ultimately leading to dramatic asymmetric introgression of ‘native’ genes into the invasive gene pool. Such introgression is predicted to be especially pronounced for organellar markers based simply on differences in effective population size and associated sensitivity to neutral drift [13,28,30].

These demographic processes may be at the heart of the observed spread of *C. maenas* mitochondrial haplotypes in the northwest Atlantic. Both southern and northern lineages are invasive in the region, but the former had been established nearly 200 years before the latter and appeared to have reached a stable range limit near Halifax, NS [18,19]. Contact between the two invasion fronts could thus reasonably be modelled as an encounter between a (southern) native and (northern) invasive population, as in Currat *et al.* [13]. Genetic introgression from south to north would thus be driven both by initial imbalance in population size at the invasion front and by rapid subsequent demographic expansion of the northern population, as the latter became abundant throughout the Canadian Maritimes within two decades after initial introduction [19]. This demographically driven introgression of mitochondrial genomes from southern to northern populations would relax demands on previous models to account for northward mitochondrial expansion through larval dispersal, presumably enabling incorporation of *L*_*diff*_ values more consistent with regional circulation statistics. In addition, the demographic models invoked here may help explain why the southern population failed to expand beyond its northern range limit prior to the second, northern invasion. If substantial northward larval transport is unnecessary to explain the bidirectional spread of mitochondrial haplotypes, then it seems likely that previous range limits were set largely by the lack of sufficient northward dispersal to establish stable populations in northern Nova Scotia, consistent with observations of ephemeral populations north of Halifax prior to the northern invasion [19]. Pringle *et al.* [21] proposed that Allee effects may have prevented establishment of crabs beyond that previous range limit until occupation of northern habitats by the more recently invading population, and our findings support this hypothesis. In effect, the arrival of a new and abundant population in the north allowed alleles from the naturalized nineteenth century population to expand beyond their previous, demographically imposed range limit, eventually reaching the provinces of Prince Edward Island, Quebec, and even Newfoundland. This ‘genetic stickiness’ in newly occupied sites was facilitated by demographic imbalances in the zone of contact, allowing southern alleles to expand northward much more rapidly than dispersal alone would have allowed.

Although there has been considerable recent interest in the implications of demographic processes for genetic dynamics at range margins, our understanding of these dynamics remains imperfect [31]. Rapid introgression of mitochondrial haplotypes relative to nuclear backgrounds is a common observation [28–30,32–35], and a number of factors may drive these patterns. For instance, the effects of demographic imbalances on mitochondrial introgression may be affected by sex-biased reproductive dynamics. In cases of highly disparate relative abundances, female mate discrimination may drive the capture of maternally inherited genomes by the more abundant population; in such circumstances, female choice and male competition renders matings by rare males unlikely, whereas rare females will be likely to accept interpopulational fertilizations [33,36,37]. Introgression is further facilitated in such cases because first generation hybrids are more likely to mate with members of the more abundant population [35]. Studies of *C. maenas* reproductive behaviour do suggest an important role for female mate choice and male competition [38], and a recent analysis of interspecific hybridization between *C. maenas* and its sibling species *C. aestuarii* concluded that these drivers best explain the observed introgression of mitochondrial genomes across species boundaries [39]. Such female-biased reproductive behaviour could further exaggerate introgression of mitochondrial genomes relative to nuclear background.

Advective systems are complex, with multiple mechanisms (i.e. larval dispersal, demographic imbalances and the potential for sex-biased reproduction) likely to be shaping the temporal development of genetic clines. In our study system, *C. maenas* shows biased introgression of mitochondrial genomes. This pattern occurs in both directions, but is most dramatic from south to north. Such introgression may be driven by different processes in different areas (or at different times) depending on demographic dynamics. Without additional data, particularly from sample sites within the initial contact zone and closer to the time of first contact, it is difficult to say for certain which models are the most appropriate for fully understanding admixture dynamics in *C. maenas*. Yet we do believe it is clear that existing knowledge of invasion history and observed genetic patterns are most consistent with selectively neutral drivers of introgression. Cytonuclear discordance has sometimes been attributed to selection on mitochondrial-encoded genes [32], as mitochondrial genes play central roles in oxidative phosphorylation, and the bioenergetic consequences of mitochondrial function may confer selective advantages for traits ranging from life history to thermal tolerance [34,40]. Among intertidal species, in particular, thermal adaptation is frequently driven by selection on mitochondrial genomes [41]. In fact, recent studies have revealed that there may be phenotypic differences between *C. maenas* populations that potentially confer selective advantage, including significantly higher physiological tolerance to low temperature stress among northern lineages in the northwest Atlantic [42,43]. Nevertheless, selective explanations are difficult to square with the bi-directional character of the mitochondrial introgression; it seems especially unlikely, for instance, that northern mitochondrial genes would be advantageous in the southern portion of the range, whereas southern genes are favoured in the north. Still, the potential role of adaptation clearly deserves future scrutiny. In particular, it has yet to be determined whether there may be some adaptive benefits to admixture that further drives evolutionary dynamics in *C. maenas*. For example, a number of studies have indicated that admixture may promote colonization and population expansion by facilitating response to novel selective pressures [44–46]. To date, observed *C. maenas* expansion patterns in the northwest Atlantic have been attributed entirely to neutral mechanisms [20,21], but this should not be taken as a reason to rule out the possibility for adaptive consequences of admixture in this system.

Beyond providing insights into the invasion history of *C. maenas* in the northwest Atlantic, our results may have more general implications for studies aimed at better appreciating the role of dispersal at population range margins. Some authors have recently attempted to estimate such dispersal by analysing neutral genetic cline decay across dynamic admixture zones [8,10]. These studies represent significant methodological advances and offer novel possibilities for indirect estimation of larval dispersal in certain systems. Our findings serve as an important reminder that dispersal may not be the only factor shaping genetic dynamics, particularly at advancing or interacting range margins. Demographic processes can have strong and varied impacts on the spatio-temporal evolution of genetic clines, and if not accounted for, may have unforeseen influences on dispersal estimates. At the edge of species ranges in particular, demographic factors and processes such as gene surfing and genetic stickiness can result in mean displacement of genes outpacing mean larval dispersal. Better appreciation of the demographic factors driving selectively neutral shifts in allele frequency could substantially improve future models of dispersal dynamics at population range limits.

## Supplementary Material

Supplemental Data File contains all data used in the analyses included in this manuscript

## Data accessibility

All genetic data are included in the electronic supplementary material, file D1.
